# Supplementary material for: Vitamin D upregulates the macrophage complement receptor immunoglobulin in innate immunity to microbial pathogens
Source: Commun Biol. 2021 Mar 25;4:401. doi: 10.1038/s42003-021-01943-3 (PMC7994403; doi:10.1038/s42003-021-01943-3)
Supplement: Supplementary file 2 — Supplementary Information [file 42003_2021_1943_MOESM2_ESM.pdf]

Supplementary Information for

Vitamin D upregulates the macrophage complement receptor immunoglobulin in innate immunity to microbial pathogens

Annabelle G Small, Sarah Harvey, Jaspreet Kaur, Trishni Putty, Alex Quach, Usma Munawara, Khalida Perveen, Andrew McPhee, Charles S Hii, Antonio Ferrante*

*Corresponding author. Email: antonio.ferrante@adelaide.edu.au

This file includes:

Supplementary Figure 1. Uncropped Western blots used throughout the manuscript

Supplementary Figure 2. The effect of anti-CRIg antibody upon phagocytosis and CR3 expression of 1,25D-treated MDM

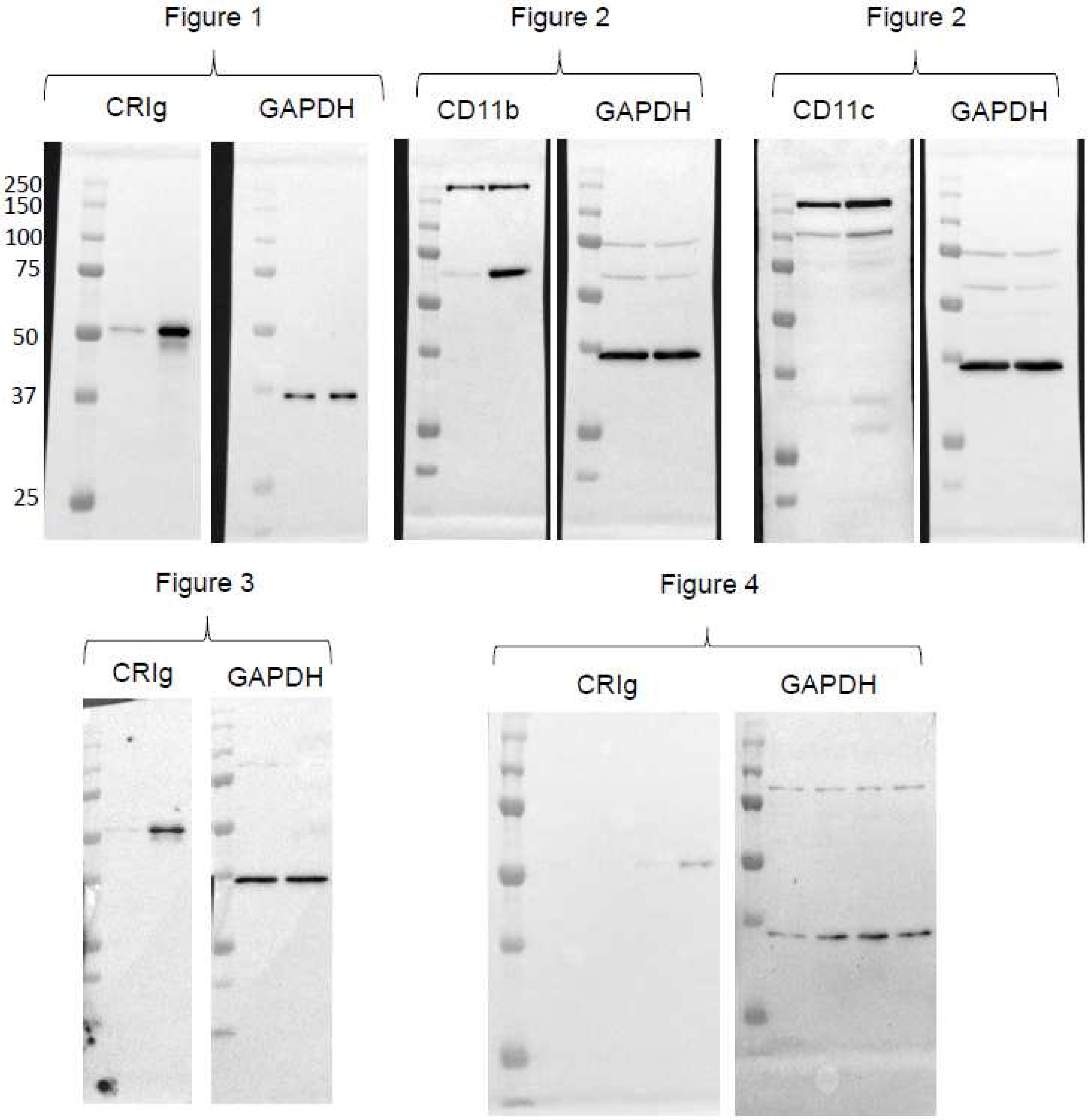

Supplementary Figure 1. Uncropped Western blots used throughout the manuscript. CRIg, CD11b, CD11c and the corresponding GAPDH blots with lanes in their entirety are presented. Precision Plus Protein™ Standards (Bio-Rad) were used for determining the molecular weights of proteins in kilodaltons (kDa).

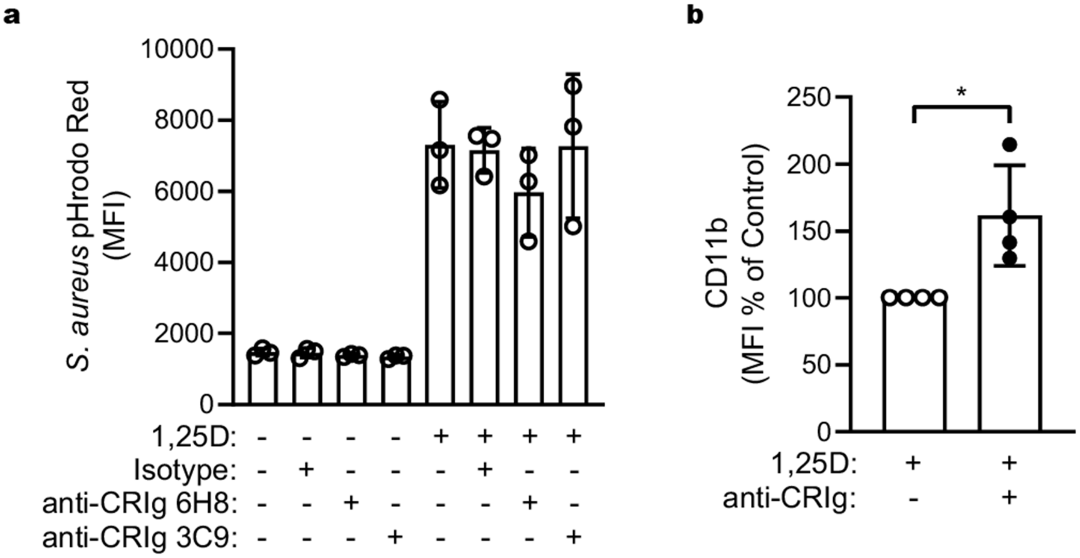

Supplementary Figure 2. The effect of anti-CRIg antibody upon phagocytosis and CR3 expression of 1,25D-treated MDM. a, Effects of adding CRIg blocking antibody on phagocytosis in 1,25D treated macrophages. MDM were treated with 100 nM of 1,25D or diluent for 24 h and then examined for phagocytosis of opsonised bacteria. The effect of blocking interaction with CRIg was achieved by adding anti-CRIg monoclonal antibodies either clone 6H8 or 3C9. **b,** The effects of anti-CRIg antibody (clone 6H8) on CR3 (anti-CD11b antibody) expression on MDM treated with 1,25D. Results are presented as mean ± s.d. of 3-4 different experiments. *P* value was calculated by the paired, two-tailed Student's *t*-test. Significance of differences between the different treatments are shown, **P* <0.05.
