# Supplementary material for: Vitamin D upregulates the macrophage complement receptor immunoglobulin in innate immunity to microbial pathogens
Source: Commun Biol. 2021 Mar 25;4:401. doi: 10.1038/s42003-021-01943-3 (PMC7994403; doi:10.1038/s42003-021-01943-3)
Supplement: Supplementary file 5 — Reporting Summary [file 42003_2021_1943_MOESM5_ESM.pdf]

nature research

Corresponding author(s): Prof. Antonio Ferrante

Last updated by author(s): Feb 16, 2021

Reporting Summary

Nature Research wishes to improve the reproducibility of the work that we publish. This form provides structure for consistency and transparency in reporting. For further information on Nature Research policies, see our Editorial Policies and the Editorial Policy Checklist.

Statistics

For all statistical analyses, confirm that the following items are present in the figure legend, table legend, main text, or Methods section.

n/a	Confirmed	
☐	☒	The exact sample size (*n*) for each experimental group/condition, given as a discrete number and unit of measurement
☐	☒	A statement on whether measurements were taken from distinct samples or whether the same sample was measured repeatedly
☐	☒	The statistical test(s) used AND whether they are one- or two-sided<br>*Only common tests should be described solely by name; describe more complex techniques in the Methods section.*
☒	☐	A description of all covariates tested
☒	☐	A description of any assumptions or corrections, such as tests of normality and adjustment for multiple comparisons
☐	☒	A full description of the statistical parameters including central tendency (e.g. means) or other basic estimates (e.g. regression coefficient) AND variation (e.g. standard deviation) or associated estimates of uncertainty (e.g. confidence intervals)
☐	☒	For null hypothesis testing, the test statistic (e.g. *F*, *t*, *r*) with confidence intervals, effect sizes, degrees of freedom and *P* value noted<br>*Give P values as exact values whenever suitable.*
☒	☐	For Bayesian analysis, information on the choice of priors and Markov chain Monte Carlo settings
☒	☐	For hierarchical and complex designs, identification of the appropriate level for tests and full reporting of outcomes
☒	☐	Estimates of effect sizes (e.g. Cohen's *d*, Pearson's *r*), indicating how they were calculated

Our web collection on statistics for biologists contains articles on many of the points above.

Software and code

Policy information about availability of computer code

Data collection	FACSCanto I was used to acquire data and FlowJo 10.1 was used to analyse the data. For Western blots, protein bands on the membranes were visualised by a ChemiDoc XRS+ Imaging System and quantitated using Image Lab Software, Version 3.0. qPCRs were generated and analysed using the Bio-Rad iQ5 optical system software version 2.1.
Data analysis	FlowJo 10.1, GraphPad Prism 8, Bio-Rad iQ5 optical system software version 2.1, Image Lab Software, Version 3.0.

For manuscripts utilizing custom algorithms or software that are central to the research but not yet described in published literature, software must be made available to editors and reviewers. We strongly encourage code deposition in a community repository (e.g. GitHub). See the Nature Research guidelines for submitting code & software for further information.

Data

Policy information about availability of data

All manuscripts must include a data availability statement. This statement should provide the following information, where applicable:

- Accession codes, unique identifiers, or web links for publicly available datasets
- A list of figures that have associated raw data
- A description of any restrictions on data availability

All data generated or analysed in this study are included either in the manuscript and supplemental data files. All raw data that supported the findings of this study are available from the corresponding author upon reasonable request.

Field-specific reporting

Please select the one below that is the best fit for your research. If you are not sure, read the appropriate sections before making your selection.

☒ Life sciences ☐ Behavioural & social sciences ☐ Ecological, evolutionary & environmental sciences

For a reference copy of the document with all sections, see nature.com/documents/nr-reporting-summary-flat.pdf

Life sciences study design

All studies must disclose on these points even when the disclosure is negative.

Sample size	No statistical methods were used to predetermine sample size.
Data exclusions	No data was excluded from the analysis.
Replication	All findings were successfully replicated in at least three independent experiments performed under identical conditions. Findings were replicated by multiple different users.
Randomization	The samples were not randomised. Single individuals were used for treatment conditions along with a matched untreated sample from the same donor. Different individuals were used for biological replicates.
Blinding	Blinding was not performed.

Reporting for specific materials, systems and methods

We require information from authors about some types of materials, experimental systems and methods used in many studies. Here, indicate whether each material, system or method listed is relevant to your study. If you are not sure if a list item applies to your research, read the appropriate section before selecting a response.

Materials & experimental systems

n/a	Involved in the study	
☐	☒	Antibodies
☒	☐	Eukaryotic cell lines
☒	☐	Palaeontology and archaeology
☒	☐	Animals and other organisms
☐	☒	Human research participants
☒	☐	Clinical data
☒	☐	Dual use research of concern

Methods

n/a	Involved in the study	
☒	☐	ChIP-seq
☐	☒	Flow cytometry
☒	☐	MRI-based neuroimaging

Antibodies

Antibodies used	The mouse monoclonal antibody (clone 3C9, for flow cytometry, 0.2 µg; for Western blotting, 1:3000) that recognizes the IgV domain of human CRIg was kindly provided by Dr. Menno van Lookeren Campagne (Genentech, San Francisco, CA). The rabbit recombinant monoclonal anti-CD11b antibody (ab133357, clone EPR1344, 1:1,000), and mouse IgG1 isotype control antibody (ab37355) were purchased from Abcam. The mouse monoclonal anti-CD11c antibody (clone N-19, 1:1,000) and goat PEconjugated anti-mouse IgG antibody were purchased from Santa Cruz Biotechnology. The mouse monoclonal anti-GAPDH (clone 71.1, 1:20,000) was obtained from Sigma-Aldrich. The polyclonal HRP-conjugated rabbit anti-mouse (P0260), anti-goat (P0449), and goat anti-rabbit (P0448) immunoglobulin antibodies (1:2000) were obtained from Dako.
Validation	Antibodies against CRIg were titrated prior to experimental runs. Goat anti-mouse IgG secondary (sc-3738, Santa Cruz), mouse anti-human GAPDH (clone 71.1, Sigma-Aldrich), anti-CD11b (ab133357, Abcam) and anti-CD11c (N-19, Santa Cruz) were validated for use in by the manufacturers; further data are available on the manufacturer's website. Secondary HRP-conjugated anti-mouse IgG, anti-goat IgG or anti-rabbit IgG were validated by Dako, Glostrup, Denmark. Anti-CRIg clone 3C9 was validated by the suppliers at Genentech.

Human research participants

Policy information about studies involving human research participants

Population characteristics	All human participants included in this study were adult healthy donors of mixed ages and genders.
Recruitment	Venous blood was collected from healthy adult volunteers under guidelines and approval of the Women's and Children's Health Network Human Research Ethics Committee. Written informed consent was obtained from all participants.

Ethics oversight	Women's and Children's Health Network Human Research Ethics Committee, HREC/15/WCHN/21

Note that full information on the approval of the study protocol must also be provided in the manuscript.

Flow Cytometry

Plots

Confirm that:

☒ The axis labels state the marker and fluorochrome used (e.g. CD4-FITC).

☒ The axis scales are clearly visible. Include numbers along axes only for bottom left plot of group (a 'group' is an analysis of identical markers).

☒ All plots are contour plots with outliers or pseudocolor plots.

☒ A numerical value for number of cells or percentage (with statistics) is provided.

Methodology

Sample preparation	Macrophage cultures were prepared into single cell suspensions in PBS and blocked using normal human IgG (Kiovig, Baxter, Old Toongabbie, NSW, Australia) prior to staining protocol detailed in the manuscript.
Instrument	FACSCanto I
Software	Flow cytometry data was captured using FACSDiva software. Results analysed using FlowJo 10.1 software.
Cell population abundance	Cell sorting was not performed.
Gating strategy	Gating strategy used is shown in figure 1e. Gates were set to Fluoresence-1 (FMO controls).

☒ Tick this box to confirm that a figure exemplifying the gating strategy is provided in the Supplementary Information.
